# Sustained impact of nosocomial-acquired spontaneous bacterial peritonitis in different stages of decompensated liver cirrhosis

**DOI:** 10.1371/journal.pone.0220666

**Published:** 2019-08-02

**Authors:** Markus Kimmann, Tammo Lambert Tergast, Marie Schultalbers, Hans Laser, Svetlana Gerbel, Michael Peter Manns, Markus Cornberg, Benjamin Maasoumy

**Affiliations:** 1 Department of Gastroenterology, Hepatology and Endocrinology, Hannover Medical School, Hannover, Germany; 2 Centre for Information Management (ZIMT), Hannover Medical School, Hannover, Germany; 3 German Centre for Infection Research (Deutsches Zentrum für Infektionsforschung DZIF), Partner-site Hannover-Braunschweig, Hannover, Germany; 4 Centre for Individualised Infection Medicine (CIIM), Hannover, Germany; Nihon University School of Medicine, JAPAN

## Abstract

**Background & aims:**

Bacterial infections, in particular a spontaneous bacterial peritonitis (SBP), are a major threat in patients with liver cirrhosis. Recently, it has been shown that the impact on mortality might be underestimated by established risk-scores. Onset of infection was suggested to define a distinct stage of cirrhosis. However, it remains unclear whether all stages of decompensated cirrhosis are equally affected. Moreover, if there is such a distinct stage, it must be determined whether it is reversible after the infection has resolved.

In this study we aimed to further analyze the impact of a current as well as a resolved SBP in different stages of decompensated liver cirrhosis.

**Methods:**

A number of 579 patients with liver cirrhosis and ascites were included. MELD-score was used to determine the stage of liver disease. Low (<15), intermediate (15–25) and high (>25) MELD-groups were compared. Patients were followed up for 90 days. Primary endpoint was overall mortality. Statistical analyses were performed using the log-rank test, Cox regression and competing risk analysis.

**Results:**

Mortality was significantly higher in patients with nosocomial-acquired SBP (nSBP) compared to patients without SBP (p<0.001;HR = 2.05). However, the most prominent difference in mortality was documented in the intermediate MELD-group (nSBP: p = 0.02;HR = 2.10). Importantly, mortality in nSBP patients remained increased even after the initial nSBP episode had resolved (p<0.01;HR = 1.90). Again, this was only significant in those with intermediate MELD-scores (p = 0.02;HR = 2.28). While a current as well as a resolved nSBP were significantly linked to a higher mortality, neither of them did increase the likelihood for liver transplantation.

**Conclusions:**

Development of nSBP is independently associated with increased mortality supporting the concept of a distinct status of cirrhosis. Importantly, the prognosis remains unfavorable even after resolution of nSBP. This could be particularly relevant for patients with intermediate MELD-scores, who have limited chances for a donor liver.

## Introduction

A particular threat for patients with liver cirrhosis is the development of bacterial infections [1]. Patients with decompensated liver cirrhosis may suffer from significant alterations of various parts of the immune system, which cause a complex immune dysfunction (cirrhosis-associated immune dysfunction; CAID) [2]. As a result, these patients have a higher susceptibility for infections but at the same time can show a hyperinflammatory response as soon as an infection has been acquired [3,4]. The most frequent type of infection in patients with decompensated liver cirrhosis is a spontaneous bacterial peritonitis (SBP) [4]. SBP frequently leads to hepatic encephalopathy (HE), variceal bleeding as well as acute kidney injury [1]. Mortality is high, in particular if the infection is acquired during hospitalization (nosocomial-acquired SBP; nSBP). Up to 30% of patients die within one month [1,5]. Some recent publications suggested that the impact of bacterial infections in cirrhosis might have been underestimated in the past i.e. when establishing scoring systems to predict short-term survival. Bacterial infections were recently identified as an independent, additional risk factor for mortality in patients with acute-on-chronic liver failure (ACLF) [6,7]. Moreover, in a landmark study by Dionigi et al. it was shown that the natural history of liver cirrhosis might be significantly accelerated after the onset of infections [8]. Of note, they could demonstrate that development of bacterial infections in patients with liver cirrhosis lead to a substantial impairment of survival, which was not sufficiently represented by the Model for End-Stage liver disease (MELD)-score. In the past, the MELD-score has been shown to overall correlate well with three-month survival in patients with decompensated liver cirrhosis [9,10]. It serves as a main criteria to rank patients for liver transplantation in the Eurotransplant region (e.g. including Germany, Austria and the Netherlands) [11]. Based on their retrospective results Dionigi et al. concluded that development of infection should be considered as a distinct stage of cirrhosis defining patients as “critical ill cirrhotics”, which might require special attendance during donor liver allocation [8]. However, more data are certainly needed (e.g. from other independent cohorts) before any adaption of criteria for liver allocation can be discussed. Furthermore, it still remains unclear whether all stages of decompensated cirrhosis are equally affected. Finally, if there is such a particular clinical status, it must be determined whether this status is reversed in those who resolve the infection.

This study aimed to further analyze the impact of bacterial infections in cirrhosis particularly focusing on SBP as the most frequent and relevant infection in these patients. First, we planned to confirm an independent impact of SBP on mortality, analyzing whether it may not be sufficiently represented by the MELD-score. Moreover, we aimed to analyze in more detail whether all stages of decompensated liver disease are affected equally by the detrimental effects of SBP. Finally, we wanted to investigate whether a negative impact on survival is sustained after the initial SBP episode has resolved, which would certainly support the thesis of a distinct clinical status in patients with cirrhosis that might require a specific management.

## Materials and methods

### Study cohort and inclusion/exclusion criteria

**Fig 1:**
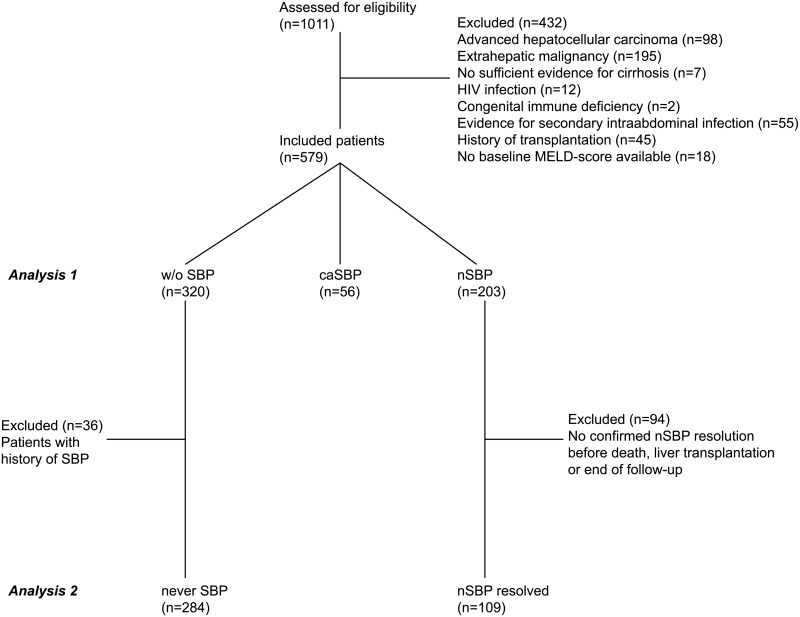
Recruitment of the study cohort, study design and distribution of the included patients into the different groups.

Patients were recruited from the well-defined Hannover Ascites Cohort, for which more than 1011 patients were evaluated. The Hannover Ascites Cohort considered all consecutive patients with liver cirrhosis who were hospitalized between January 2012 and June 2016 at Hannover Medical School and underwent at least one paracentesis during the time of hospitalization. These patients were automatically identified retrospectively by the clinical data warehouse of Hannover Medical School, as described previously [12,13]. A careful manual review of each individual patient file was carried out to validate the automatic selection of patients. The following exclusion criteria were applied: Evidence for secondary intraabdominal infection, presence of malignancy (except for hepatocellular carcinoma within the MILAN criteria), peritonitis carcinomatosa, infection with the human immunodeficiency virus (HIV), history of solid organ or stem cell transplantation and congenital immune dysfunction. In addition, all patients without evidence of liver cirrhosis were excluded. Most patients were hospitalized due to hepatic decompensation (S1 Table). For the current study, all patients, in whom MELD-score was not available at baseline (±7 days) were also excluded. Overall, 579 patients were eligible (Fig 1).

The retrospective analysis of anonymized patients´ data was approved by the local ethics committee and data protection officer of Hannover Medical School. The study was carried out according to the Declaration of Helsinki.

### Data assessment

Clinical and laboratory data were collected from patients´ files. MELD-score was calculated based on the available laboratory values that were closest to baseline (maximum ±7 days). All other laboratory values were assessed within 48 hours of baseline. Paracentesis was performed to diagnose SBP and to determine SBP resolution. SBP was diagnosed in patients with ≥ 500 nucleus containing cells/mm^3^ ascites fluid. [12–14]. SBP that occurred within the first 48 hours of hospitalization was classified as a community-acquired SBP (caSBP). Development of SBP more than 48 hours after hospital admission was considered as a nosocomial-acquired infection (nSBP) [1,15]. Diagnosis of liver cirrhosis was made on the basis of either ultrasound, FibroScan (≥ 14.5 kPa), typical biochemical results (i.e. AST/ALT ratio >1, bilirubin >1.5 x ULN, platelets < 100 (10^3^/μl), Albumin < 35 g/L) and/ or liver biopsy (F4 in Metavir or F5-6 in ISHAK) or (in the majority of cases) a combination of the above [14,16]. ACLF was diagnosed in concordance with the current recommendations of the European Association for the Study of the Liver (EASL) at baseline. In short, occurrence of renal failure alone or any other single organ failure with presence of cerebral dysfunction and/or renal dysfunction defines ACLF grade one. Organ failure(s) were determined using the CLIF-SOFA Score [3,17,18].

### Study design

Two main analyses were performed in the framework of this study:

In the first analysis the impact of a current SBP episode was studied. Overall, 579 patients were eligible and distributed to one of the following three groups (Fig 1):

1 *Without (w/o) SBP*: patients without any SBP episode during the 90-day follow-up period (n = 320)
2 *nSBP*: patients, in whom SBP occurred more than 48 hours after hospital admission (n = 203)
3 *caSBP*: patients with a caSBP at baseline or onset <48 hours after hospital admission (n = 56)

The follow-up period was 90 days (median follow-up 66 days) and started (baseline) at the time of the first paracentesis (w/o SBP) and the time of SBP diagnosis (nSBP, caSBP), respectively.

Additionally, we compared the mortality between patients with and without ACLF at baseline. We also compared the mortality between patients of the w/o SBP and nSBP group with and without ACLF at baseline.

The second analysis focused on the impact of a resolved nSBP episode on mortality during further follow-up. Based on the w/o SBP and the nSBP cohort the following two groups were defined (Fig 1):

4 *never SBP*: patients from the w/o SBP group without any previous SBP in the past (n = 284).
5 *nSBP resolved*: patients from the nSBP group in whom a follow-up paracentesis during the 90-day follow-up proofed complete resolution of nSBP (n = 109). The date on which the nSBP resolved was used as a new baseline for these patients.

The follow-up period was 90 days (median follow-up 70 days) and started (baseline) at the time of the first paracentesis (never SBP) and nSBP resolution paracentesis (nSBP resolved), respectively.

Primary endpoint of the study was overall mortality within 90 days. Follow-up ended with death or liver transplantation.

### Statistics

Statistical analyses were performed with SPSS (Version 25.0; IBM, New York, USA), Microsoft Excel (Microsoft, Redmond, Washington, USA), GraphPad Prism (version 7.0; GraphPad Software Inc. La Jolla, California, USA) and R (R.app GUI 1.70 (7612 El Capitan build), S. Urbanek & H.-J. Bibiko, R Foundation for Statistical Computing). Categorical variables are presented as number and percentage, while continuous variables are presented as median with interquartile range. Continuous variables were analyzed using the Mann-Whitney-U-test, while Pearson´s Chi square test was used for categorical variables.

Primary endpoint of the study was overall mortality within 90 days. Patients who did not reach the end point (death) during follow-up were censored as alive at the last follow-up contact or at time of liver transplantation during follow-up, respectively. Curves depicting mortality were generated and further analyzed with the log-rank test to visualize whether there was a difference in terms of mortality between the different groups and subgroups. To control for relevant confounders of mortality different uni- and multivariate Cox regression models were used. In all Cox regression models age and gender were included as well as baseline parameters considered to be relevant indicators for the severity of liver disease. Selected indicators for liver disease severity were MELD (liver synthesis and detoxification as well as renal function), platelets and sodium (portal hypertension) as well as ALT (x ULN) and GGT (x ULN) (ongoing hepatic injury). Depending on the specific analysis additional parameters were considered:

*Analysis 1*:
  - Model 1: When analyzing the cohort of nSBP and w/o SBP patients (analysis 1) the presence of nSBP was added to the Cox regression analysis.
*Analysis 2*:
  - Model 2: When analyzing the cohort of nSBP resolved and never SBP patients (analysis 2) “resolved nSBP” was added to the Cox regression analysis.
  - Model 3: When analyzing the subgroup of nSBP resolved patients (analysis 2) an additional parameter for the Cox regression analysis was intake of secondary antibiotic prophylaxis.

All parameters having p-values <0.1 in the univariate analysis were considered as potential risk factors for the death and therefore included in the multivariate model. The multivariate Cox regression analysis was performed using backwards stepwise logistic regression, excluding all parameters with p-values >0.1. In case of missing parameters within the Cox regression model the case was excluded for this particular analysis.

In addition, to compare the likelihood of death and liver transplantation between nSBP and w/o SBP patients as well as nSBP resolved and never SBP patients, we performed a competing risk analysis [19]. Death and liver transplantation were treated as competing risks. Patients who neither reached the end point death nor liver transplantation during follow-up were censored as alive at the last follow-up contact.

## Results

### Study cohort

Overall, 579 patients were included. Median age of the patients was 56 years, the majority was male (62.00%) and median MELD-score was 18.54. A number of 239 patients (41.28%) had ACLF at baseline (Table 1). There were some significant differences in terms of baseline parameters between the caSBP, nSBP and w/o SBP groups. Leukocytes, MELD-score, INR, creatinine, etiology of cirrhosis, history of SBP, HE, ACLF and CRP were different between nSBP and w/o SBP patients (Table 1). Significant differences between the caSBP and the w/o SBP group included gender distribution, age, sodium and ALT. MELD-score was numerically lower, while platelet count was higher in the caSBP compared to the w/o SBP group.

**Table 1:** Baseline characteristics of the study cohort (analysis 1).

	Overall cohort (n = 579)	w/o SBP (n = 320)	caSBP (n = 56)	nSBP (n = 203)	p-value
**Age (years)**	56.00 (48.00–63.00)	55.00 (48.00–62.00)	58.50 (49.25–70.00)	56.00 (49.00–63.00)	0.48
**Gender (female/male), n (%)**	220/359 (38.00/62.00)	135/185 (42.19/57.81)	10/46 (17.86/82.14)	75/128 (36.95/63.05)	0.23
**History of SBP, n (%)**	82 (14.16)	36 (11.25)	6 (10.71)	40 (19.70)	<0.01
**Etiology of cirrhosis**					
**ASH, n (%)**	298 (51.47)	179 (55.94)	29 (51.79)	90 (44.33)	0.01
**Viral, n (%)**	101 (17.44)	54 (16.88)	9 (16.07)	38 (18.72)	0.59
**Other, n (%)**	214 (36.96)	104 (32.50)	22 (39.29)	88 (43.35)	0.02
**MELD**	18.54 (13.91–25.03)	17.88 (12.66–24.46)	16.88 (12.89–24.09)	20.28 (14.68–27.78)	<0.01
**Creatinine (mg/dl)**	1.30 (0.90–2.04)	1.22 (0.83–1.92)	1.24 (0.90–2.00)	1.44 (1.02–2.25)	<0.01
**Bilirubin (mg/dl)**	2.51 (1.11–7.31)	2.46 (1.17–6.71)	2.43 (1.05–5.06)	3.10 (1.11–9.18)	0.28
**INR**	1.46 (1.29–1.72)	1.41 (1.28–1.66)	1.43 (1.27–1.67)	1.52 (1.32–1.91)	<0.01
**Sodium (mmol/l)**	136.00 (132.00–139.00)	136.00 (133.00–139.00)	135 (131.50–137.00)	135.00 (130-00-139.00)	0.06
**ALT (x ULN)**	0.71 (0.44–1.24)	0.71 (0.47–1.21)	0.51 (0.36–0.90)	0.76 (0.45–1.33)	0.72
**GGT (x ULN)**	2.15 (1.04–4.41)	2.16 (1.05–4.74)	2.30 (1.17-3-32)	2.11 (0.98–4.05)	0.46
**Platelets (10^3^/μl)**	107.00 (68.00–170.00)	112.00 (70.75–174.00)	127.00 (78.50–198.00)	99.00 (62.00–158.00)	0.06
**Leukocytes (10^3^/μl)**	7.60 (4.88–12.10)	7.00 (4.58–10.40)	8.10 (5.35–14.45)	8.70 (5.80-13-40)	<0.001
**CRP (mg/l)**	30.00 (13.55–56.75)	24.50 (12.00–47.00)	24.00 (12.00–60.50)	38.00 (18.00–74.45)	<0.001
**HE, n (%)**	129 (22.28)	54 (16.88)	14 (25.00)	61 (30.04)	<0.01
**ACLF, n (%)**	239 (41.28)	117 (36.56)	24 (42.86)	98 (48.28)	<0.01
**Esophageal varices, n (%)**	419 (72.37)	231 (72.19)	37 (66.07)	151 (74.38)	0.58
**History of variceal bleeding, n (%)**	79 (13.64)	45 (14.06)	8 (14.29)	26 (12.81)	0.68

P compares patients with nSBP and w/o SBP. Mann-Whitney-U-test was used for continuous parameters, Chi-square test for categorical parameters. Continuous parameters are shown as median with interquartile range. Mixed etiology of cirrhosis: w/o SBP (n = 17), caSBP (n = 4) and nSBP (n = 14). nSBP: nosocomial-acquired spontaneous bacterial peritonitis; caSBP: community-acquired spontaneous bacterial peritonitis; w/o SBP: without spontaneous bacterial peritonitis; HE: Hepatic encephalopathy.

### Overall impact of SBP on mortality

Mortality was similar between caSBP and w/o SBP patients (p = 0.46; S1 Fig). In contrast, we documented a significantly higher mortality in patients with nSBP as compared to w/o SBP patients (p<0.001; HR = 2.05; S1 Fig). MELD-score (p<0.001; adjusted HR = 1.12) and nSBP (p<0.01; adjusted HR = 1.68) were independent risk factors for death in the multivariate model (Table 2).

**Table 2:** Risk factors for death in w/o SBP and nSBP patients.

Risk factors for death	Univariate HR	95% CI	p-value	Multivariate Adjusted HR	95% CI	p-value
**MELD**	1.13	1.10–1.15	**<0.001**	1.12	1.10–1.15	**<0.001**
**nSBP (yes)**	2.06	1.41–3.01	**<0.001**	1.68	1.15–2.46	**<0.01**
Age (years)	1.01	0.99–1.02	0.53			
Gender (Male)	1.21	0.82–1.78	0.33			
ALT (x ULN)	1.02	0.99–1.05	0.20			
GGT (x ULN)	0.998	0.95–1.05	0.95			
Platelets (10^3^/μl)	0.997	0.995–1.000	0.04	1.00	0.998–1.002	0.65
Sodium	0.98	0.95–1.01	0.16			

Uni- and multivariate Cox-regression analysis (Model 1) to identify independent risk factors for death in patients with nSBP and w/o SBP (all MELD-Scores). nSBP: nosocomial-acquired spontaneous bacterial peritonitis; w/o SBP: without spontaneous bacterial peritonitis; CI: confidence interval; HR: Hazard Ratio.

Presence of ACLF was associated with an increased mortality in the overall cohort (p<0.001; HR = 3.48; S2 Fig). Even in the cohort of ACLF patients development of a nSBP was associated with a significantly higher mortality (p = 0.02; HR = 1.73; S3 Fig). However, in the multivariate analysis this closely failed to reach statistical significance (p = 0.057; S2 Table). Similar results were documented in those without ACLF (p = 0.02; HR = 2.08; S4 Fig and p = 0.14; S3 Table).

### Impact of nSBP on mortality in different stages of decompensated liver disease (as defined by the MELD-score)

**Fig 2:**
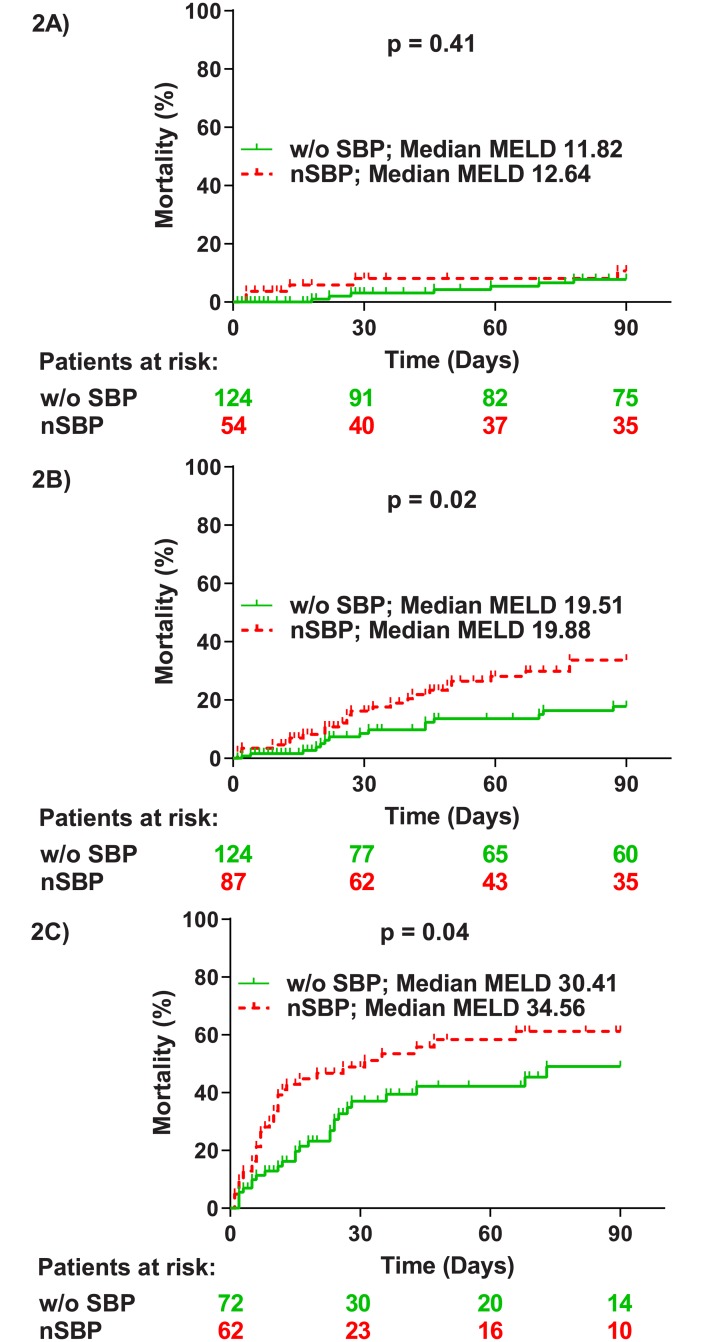
Mortality comparison between nSBP and w/o SBP patients. Mortality within 90 days from baseline in patients with nSBP and w/o SBP considering patients with low MELD-scores (below 15) (A), patients with intermediate MELD-scores (between 15 and 25) (B) and patients with high MELD-scores (over 25) (C). P-values were calculated using the log-rank test. nSBP: nosocomial-acquired spontaneous bacterial peritonitis; w/o SBP: without spontaneous bacterial peritonitis.

We further aimed to investigate whether specific stages of decompensated cirrhosis are particularly vulnerable for the detrimental effects of nSBP. Therefore, the w/o SBP and nSBP cohorts were divided according to the baseline MELD-score into three different groups. A low MELD-group (<15) considered to have relatively mild disease (n = 178; no liver transplantation was performed during follow-up; S4 Table), an intermediate MELD-group (between 15 and 25) (n = 211) considered to have severe liver disease but still relatively low chances for a donor liver (at least in the Eurotransplant region) and a high MELD-group (>25) with very advanced liver disease and decent chances for a donor liver (n = 134) (S5 Table). In the low MELD-group, no significant difference in mortality was documented between nSBP and w/o SBP patients (p = 0.41). Within the high MELD-group mortality was significantly higher in patients with nSBP (p = 0.04; HR = 1.68). However, the median MELD-score was also significantly higher in the nSBP group (nSBP: 34.56 and w/o SBP: 30.41; p<0.01). Interestingly, the most prominent difference was documented among patients with intermediate MELD-score. Mortality was significantly higher in the nSBP cohort (p = 0.02; HR = 2.10). Of note, median MELD-scores were similar between both groups (nSBP: 19.88 and w/o SBP: 19.51; p = 0.73) (Fig 2A–2C).

Multivariate Cox regression analysis including all nSBP and w/o SBP patients were performed separately for all three different MELD subgroups to identify independent risk factors for mortality. nSBP remained a statistically significant risk factor for mortality in the intermediate MELD-group after multivariate analysis (p = 0.03; adjusted HR = 2.09). Interestingly, in the high MELD-group only age (p<0.01; adjusted HR = 1.05) and MELD (p<0.001; adjusted HR = 1.16) but not nSBP (p = 0.19) were independently associated with mortality (Table 3).

**Table 3:** Risk factors for death in w/o SBP and nSBP patients.

**A (MELD <15)**						
**Risk factors for death**	Univariate HR	95% CI	p-value	Multivariate Adjusted HR	95% CI	p-value
MELD	0.92	0.72–1.18	0.52			
nSBP (yes)	1.61	0.51–5.08	0.41			
**Age (years)**	1.06	1.005–1.119	**0.03**	1.09	1.01–1.17	**0.03**
Gender (Male)	1.11	0.35–3.50	0.86			
ALT (x ULN)	1.86	0.77–4.53	0.17			
**GGT (x ULN)**	1.13	1.00–1.27	**0.05**	1.13	1.004–1.275	**0.04**
Platelets (10^3^/μl)	1.003	0.999–1.008	0.13			
Sodium	1.10	0.96–1.25	0.16			
**B (MELD 15–25)**						
**Risk factors for death**	Univariate HR	95% CI	p-value	Multivariate Adjusted HR	95% CI	p-value
**MELD**	1.12	1.01–1.24	**0.03**	1.12	1.01–1.25	**0.03**
**nSBP (yes)**	2.04	1.07–3.89	**0.03**	2.09	1.09–3.99	**0.03**
Age (years)	1.004	0.98–1.03	0.74			
Gender (Male)	1.25	0.65–2.38	0.50			
ALT (x ULN)	0.93	0.75–1.17	0.54			
GGT (x ULN)	1.01	0.93–1.10	0.73			
Platelets (10^3^/μl)	1.001	0.997–1.005	0.61			
Sodium	0.99	0.94–1.04	0.76			
**C (MELD >25)**						
**Risk factors for death**	Univariate HR	95% CI	p-value	Multivariate Adjusted HR	95% CI	p-value
**MELD**	1.17	1.10–1.23	**<0.001**	1.16	1.09–1.23	**<0.001**
nSBP (yes)	1.68	1.01–2.82	0.047	1.47	0.83–2.58	0.19
**Age (years)**	1.04	1.01–1.07	**<0.01**	1.05	1.01–1.08	**<0.01**
Gender (Male)	0.98	0.57–1.67	0.94			
ALT (x ULN)	1.05	0.995–1.098	0.08	1.04	0.99–1.09	0.15
GGT (x ULN)	0.98	0.91–1.04	0.46			
Platelets (10^3^/μl)	0.997	0.993–1.001	0.09	0.998	0.994–1.001	0.17
Sodium	1.004	0.97–1.04	0.84			

Uni- and multivariate Cox-regression analysis (Model 1) to identify independent risk factors for death in patients with nSBP and w/o SBP divided in patients with low MELD-scores (below 15) (A), patients with intermediate MELD-scores (between 15 and 25) (B) and patients with high MELD-scores (over 25) (C).

In addition, a competing risk analysis was performed to further investigate the impact of nSBP on mortality but also on the likelihood for liver transplantation. Of note, while the risk for death was significantly higher during follow-up in the nSBP patients (p<0.001), this was not accompanied by better chances to receive a liver transplantation (p = 0.39) (S5 Fig).

### Impact of a resolved nSBP on mortality in the different MELD-groups

In order to investigate whether developing a nSBP remains independently associated with an increased mortality even after the infection has resolved an additional analysis was performed (analysis 2). Only nSBP patients in whom a follow-up paracentesis proofed complete resolution of SBP (nSBP resolved) were considered for this analysis. As a control group we selected patients who never had experienced any SBP episode in the past (never SBP) (Fig 1). Creatinine and platelets were the only significantly different parameters (both p = 0.02) between both groups at baseline (Table 4).

**Table 4:** Baseline characteristics of the study subcohort for analysis 2.

	Overall subcohort analysis 2 (n = 393)	Never SBP (n = 284)	nSBP resolved (n = 109)	p-value
**Age (years)**	55.00 (48.00–62.00)	55.00 (48.00–62.00)	56.00 (48.50–62.50)	0.80
**Gender (female/male), n (%)**	162/231 (41.22/58.78)	123/161 (43.31/56.56)	39/70 (35.78/64.22)	0.18
**Etiology of cirrhosis**				
**ASH, n (%)**	213 (54.20)	159 (55.99)	54 (49.54)	0.27
**Viral, n (%)**	60 (15.27)	46 (16.20)	14 (12.84)	0.41
**Other, n (%)**	140 (35.62)	92 (32.39)	48 (44.03)	0.51
**MELD**	18.15 (12.98–24.46)	17.50 (12.64–24.42)	19.14 (13.97–25.53)	0.16
**Creatinine (mg/dl)**	1.24 (0.87–1.91)	1.17 (0.81–1.85)	1.45 (1.01–1.94)	0.02
**Bilirubin (mg/dl)**	2.51 (1.11–7.40)	2.51 (1.17–7.31)	2.40 (0.99–7.92)	0.84
**INR**	1.42 (1.28–1.66)	1.41 (1.28–1.66)	1.44 (1.32–1.66)	0.39
**Sodium (mmol/l)**	136.00 (132.00–139.50)	136.00 (133.00–139.00)	135.00 (130.00–140.00)	0.38
**ALT (x ULN)**	0.73 (0.47–1.24)	0.73 (0.47–1.24)	0.78 (0.50–1.27)	0.94
**GGT (x ULN)**	2.10 (1.00–4.69)	2.07 (1.02–4.78)	2.36 (0.96–4.65)	0.91
**Platelets (10^3^/μl)**	107.00 (68.00–169.00)	114.00 (71.25–174.75)	94.00 (63.00–153.00)	0.02
**Leukocytes (10^3^/μl)**	7.30 (4.70–10.90)	7.10 (4.60–10.78)	7.60 (5.20–11.50)	0.36
**CRP (mg/l)**	24.95 (12.00–47.00)	24.20 (12.00–48.00)	27.10 (13.50–46.00)	0.64

P compares nSBP resolved and never SBP patients. Mann-Whitney-U-test was used for continuous parameters, Chi-square test for categorical parameters. Continuous parameters are shown as median with interquartile range. Mixed etiology of cirrhosis: Never SBP (n = 13), nSBP resolved (n = 7). nSBP resolved: resolved nosocomial-acquired spontaneous bacterial peritonitis; never SBP: no history of current or past spontaneous bacterial peritonitis.

**Fig 3:**
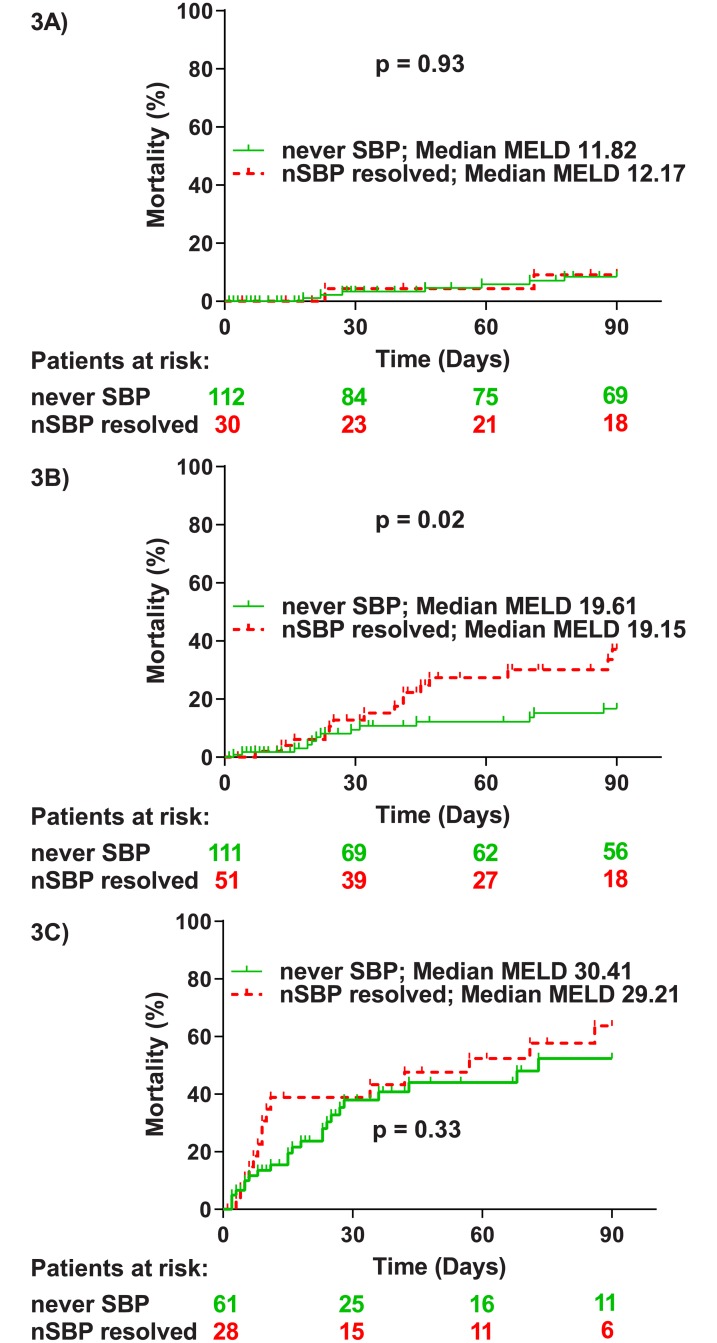
Mortality comparison between nSBP resolved and never SBP patients. Mortality within 90 days from baseline in never SBP and nSBP resolved patients considering patients with low MELD-scores (below 15) (A), patients with intermediate MELD-scores (between 15 and 25) (B) and patients with high MELD-scores (over 25) (C). P-values were calculated using the log-rank test. nSBP resolved: resolved nosocomial-acquired spontaneous bacterial peritonitis; never SBP: no history of current or past spontaneous bacterial peritonitis.

Overall, mortality was significantly higher in patients with resolved nSBP compared to the never SBP patients (p<0.01; HR = 1.90; S6 Fig). We further analyzed the impact of a resolved nSBP in different stages of decompensated liver disease: No difference in mortality was documented among patients with MELD-scores below 15 (p = 0.93) (n = 142). Of note, in patients with MELD-scores over 25 (n = 89) resolved nSBP was associated with a numerical increase in mortality (p = 0.33). However, the only significant difference was again detected in the subgroup of patients with intermediate MELD-scores (p = 0.02; HR = 2.28) (n = 162) (Fig 3A–3C).

In the multivariate analysis resolved nSBP (p = 0.02; adjusted HR = 1.73) and MELD (p<0.001; adjusted HR = 1.11) were independent risk factors for mortality in the entire cohort, while in the subgroup of patients with intermediate MELD-scores only resolved nSBP but not the MELD-score was significantly associated with higher mortality (p = 0.03; HR 2.30) (Table 5).

**Table 5:** Risk factors for death in never SBP and resolved nSBP patients.

**A (All MELD-Scores)**						
**Risk factors for death**	Univariate HR	95% CI	p-value	Multivariate Adjusted HR	95% CI	p-value
**MELD**	1.11	1.09–1.14	**<0.001**	1.11	1.08–1.14	**<0.001**
**Resolved nSBP (yes)**	1.90	1.20–3.01	**<0.01**	1.73	1.10–2.74	**0.02**
Age (years)	1.01	0.99–1.03	0.49			
Gender (Male)	1.10	0.70–1.75	0.68			
ALT (x ULN)	1.04	0.98–1.11	0.21			
GGT (x ULN)	1.02	0.97–1.06	0.44			
Platelets (10^3^/μl)	0.998	0.995–1.001	0.17			
Sodium	0.98	0.95–1.02	0.41			
**B (MELD <15)**						
**Risk factors for death**	Univariate HR	95% CI	p-value	Multivariate Adjusted HR	95% CI	p-value
MELD	0.90	0.67–1.20	0.46			
Resolved nSBP (yes)	1,07	0.22–5.15	0.93			
Age (years)	1.06	1.00–1.12	0.07	1.06	0.99–1.14	0.12
Gender (Male)	0.64	0.17–2.39	0.51			
ALT (x ULN)	1.21	0.67–2.18	0.53			
**GGT (x ULN)**	1.18	1.03–1.34	**0.01**	1.16	1.02–1.32	**0.02**
Platelets (10^3^/μl)	1.004	0.999–1.009	0.10			
Sodium	1.11	0.95–1.31	0.19			
**C (MELD 15–25)**						
**Risk factors for death**	Univariate HR	95% CI	p-value	Multivariate Adjusted HR	95% CI	p-value
MELD	1.09	0.96–1.24	0.20			
**Resolved nSBP (yes)**	2.30	1.09–4.84	**0.03**			
Age (years)	1.01	0.98–1.04	0.66			
Gender (Male)	0.95	0.45–2.01	0.89			
ALT (x ULN)	1.03	0.90–1.18	0.70			
GGT (x ULN)	1.01	0.92–1.12	0.77			
Platelets (10^3^/μl)	1.000	0.995–1.005	0.93			
Sodium	0.96	0.91–1.02	0.19			
**D (MELD >25)**						
**Risk factors for death**	Univariate HR	95% CI	p-value	Multivariate Adjusted HR	95% CI	p-value
**MELD**	1.08	1.01–1.16	**0.03**	1.08	1.01–1.16	**0.03**
Resolved nSBP (yes)	1.38	0.72–2.65	0.34			
**Age (years)**	1.06	1.02–1.10	**<0.01**	1.06	1.02–1.10	**<0.01**
Gender (Male)	1.31	0.67–2.56	0.43			
ALT (x ULN)	0.99	0.90–1.08	0.82			
GGT (x ULN)	0.99	0.94–1.05	0.80			
Platelets (10^3^/μl)	0.997	0.992–1.002	0.18			
Sodium	1.02	0.98–1.07	0.32			

Uni- and multivariate Cox-regression analysis (Model 2) to identify independent risk factors for death in the resolved nSBP and never SBP group considering all patients (A), patients with low MELD-scores (below 15) (B), patients with intermediate MELD-scores (between 15 and 25) (C) and patients with high MELD-scores (over 25) (D). resolved nSBP: resolved nosocomial-acquired spontaneous bacterial peritonitis; never SBP: no history of current or past spontaneous bacterial peritonitis; CI: confidence interval; HR: Hazard Ratio.

Additionally, we performed a competing risk analysis to investigate the impact of a resolved nSBP on the likelihood of death and liver transplantation. Importantly, while a resolved nSBP was linked to significantly higher mortality (p<0.01), incidence of liver transplantation was almost the same between the nSBP resolved and the never SBP group (p = 0.42) (S7 Fig).

#### Impact of recurrent nSBP and the role of antibiotic prophylaxis

A number of 43 patients of the nSBP resolved cohort developed a further SBP episode during 90-day follow-up. Mortality was numerically higher in the group of patients with SBP recurrence compared to the remaining nSBP resolved patients (p = 0.14; S8 Fig). Overall, 67 out of 109 nSBP resolved patients (61.47%) received a secondary antibiotic prophylaxis as part of their medication (S6 Table). Although, secondary antibiotic prophylaxis did not significantly decrease the incidence of SBP recurrence during 90-day follow-up (p = 0.38; S9 Fig) it was still associated with a significantly decreased mortality (log-rank: p<0.01; HR = 0.41; S10 Fig and multivariate Cox regression: p = 0.03; HR = 0.45; S7 Table).

## Discussion

In this large and well-defined cohort we confirmed the exceptional and independent impact of SBP on the clinical course of patients with decompensated liver cirrhosis. Of note, we were able to show for the first time that not all stages of decompensated liver disease are affected equally. The independent, negative impact of nSBP was most pronounced in the group of patients with intermediate MELD-scores (15–25), who despite the presence of advanced liver disease have limited chances for timely donor liver allocation in the Eurotransplant region. Importantly, we also demonstrated that the negative impact of nSBP on survival remains significant even in those who resolve the initial SBP episode. These data support the recent concept that considers development of severe infection such as nSBP as a distinct clinical status of cirrhosis. Our data suggest that this definition might even be extended to those with resolved SBP.

Some of our data are in line with the recent landmark study by Dionigi et al. who documented a significantly increased mortality in cirrhotic patients in case of any bacterial infections compared to non-infected individuals. Of note, the individual role of specific infections was not analyzed [8]. Bacterial infections, in general, represent a particular threat in patients with decompensated cirrhosis, as they can be associated with a significant systemic inflammatory response leading to considerable hemodynamic changes. Patients with decompensated cirrhosis are characterized by a high vulnerability to these hemodynamic disturbances due to the presence of portal hypertension, which can be further complicated by cirrhotic cardiomyopathy and/or adrenal insufficiency [20–22]. However, severity of systemic inflammation as well as the effects on portal hypertension may be influenced by the type of infection. In patients with cirrhosis the most relevant type of infection is SBP [10,23]. SBP is a specific complication of cirrhosis and associated with a high 28d-mortality rate of up to 20–30% [23]. Due to the high relevance in cirrhosis we decided to focus on SBP in our study.

One of the key novel findings of our study was that the negative impact of nSBP on mortality was most pronounced in the group of patients with intermediate MELD-scores (15–25). The better-preserved liver function, which is possibly also associated with a higher health status, might be the reason that no significant effect was observed in the group of patients with MELD-scores <15. Patients with MELD-scores >25 certainly suffer from end-stage liver disease and belong to a highly vulnerable group. However, likelihood for death or liver transplantation is high even without the presence of nSBP. In contrast, a MELD-score of 15–25 may represent a borderline situation. Chances for timely allocation of a donor liver are quite low at least in the Eurotransplant region. Without the occurrence of acute triggers or complications patients might well remain in a relatively stable clinical state. However, advanced liver cirrhosis is already present. The incidence of a nSBP may easily lead to further deterioration of hepatic function and portal hypertension altering the natural history closer towards the end-stage of the disease. This might explain that those with an intermediate MELD-score were particularly vulnerable for the detrimental effects of a nSBP.

In our cohort only nSBP but not caSBP was an independent risk factor for death after adjusting for MELD-score as well as several other indicators of the severity of liver disease. A better outcome of caSBP compared to nSBP has been reported by other centers as well [5,24], which has usually been attributed to a higher prevalence of multidrug resistant bacteria (MDRB) in nSBP patients and the consecutive failure of the initial antibiotic treatment strategy [5,25]. In our cohort MDRB were found in five patients only (all nSBP). However, it has to be considered that the responsible bacteria could be isolated in less than 40% of SBP patients, which is in line with several other studies on SBP and underlines the urgent unmet need for the development of improved diagnostic tools in this setting [26,27] (S8 and S9 Tables).

Another major finding of our study was that the impact of nSBP on mortality did not disappear even after the nSBP has completely resolved. Of note, SBP recurrence after this time point was not significantly associated with mortality during 90-day follow-up. In line with our data Dionigi et al. documented that mortality in patients with bacterial infections remains increased even after excluding those who died early within 30 days after onset of the infection [8]. Together these findings suggest that initial development of nSBP is a valuable additional predictive marker for mortality in patients with advanced liver cirrhosis that is independent from the MELD-score and has prognostic value that exceeds the short-term mortality of bacterial infection. It may rather indicate severity of liver disease possibly by reflecting cirrhosis-associated immune dysfunction and/or portal hypertension, which, importantly, are not included in the MELD-score.

Our study has some limitations that need to be considered while interpreting the data. Although patients were collected consecutively, data assessments as well as statistical analysis were performed retrospectively. SBP diagnosis was not made based on the polymorphonuclear cell count. Thus, minor differences in the patient classification might be considered when comparing our results to other studies on SBP. Moreover, data were collected at our center only. Thus, we are i.e. unable to control for regional differences in the MDRB prevalence.

However, we think that our results provide some important clinical implications. We identified individuals with intermediate MELD-scores and with current or resolved nSBP as a particularly vulnerable group of patients who require an improved management. A key part of this management is the consequent administration of a secondary antibiotic prophylaxis in patients with history of SBP. Secondary prophylaxis with norfloxacin has been proven to decrease SBP recurrence and to improve survival [28]. Therefore, it has been considered as first option for antibiotic prophylaxis by many centers in the past [29]. Of note, usage of antibiotic prophylaxis was able to improve the prognosis of patients after resolution of nSBP in our study. Contrarily, we did not document any significant difference in the SBP recurrence rate at least during the 90-day follow-up. However, except of prevention of SBP recurrence additional effects of antibiotic prophylaxis may have contributed to the decreased mortality. Potentially beneficial immunomodulatory effects of fluoroquinolones, in particular of norfloxacin, have been described in patients with liver cirrhosis [30,31]. However, with regard to SBP prevention the increasing prevalence of MDRB will make antibiotic prophylaxis more and more challenging [32]. Of note, norfloxacin is no longer available in the USA [29] and there has been a warning of the European Medicines Agency (EMA) from 5^th^ of October 2018 (EMA/668915/2018) restricting the use of fluoroquinolones to emergency cases only due to their unfavorable safety profile. Sufficient evidence for the usage of alternative antibiotics is lacking. Therefore, alternative strategies will be required in the future.

However, the only curative option for patients with advanced liver disease remains liver transplantation. Given the fact that differences in mortality between patients without SBP and nSBP patients were particularly high in those with MELD-scores between 15 and 25, might indicate that these patients could have a disadvantage in MELD based liver organ allocation systems. It remains unclear whether and how long antibiotic prophylaxis can overcome such disadvantages. Future studies are needed to further analyze whether an adaption of the criteria for donor liver allocation is needed for these patients.

In summary we showed that development of a nSBP is independently associated with increased mortality supporting the concept of a distinct status of cirrhosis. This particularly affects patients with intermediate MELD-scores, who have limited chances for a donor liver despite advanced liver disease. Importantly, the prognosis remains unfavorable in these patients even if the initial nSBP episode has resolved. For the moment, antibiotic prophylaxis helps to decrease mortality. However, alternative strategies might be required for these patients in the future.

## Supporting information

S1 Dataset Minimal data set. (XLSX)

S1 Fig Mortality comparison within the overall cohort (analysis 1). P-value was calculated using the log-rank test. p_1_ compares w/o SBP and nSBP, p_2_ compares w/o SBP and caSBP.
(DOCX)

S2 Fig Mortality comparison between patients with and without at least grade 1 ACLF within the overall cohort (analysis 1). P-value was calculated using the log-rank test.
(DOCX)

S3 Fig Mortality comparison between patients with ACLF at baseline considering w/o SBP and nSBP patients. P-value was calculated using the log-rank test.
(DOCX)

S4 Fig Mortality comparison between patients without ACLF at baseline considering w/o SBP and nSBP patients. P-value was calculated using the log-rank test.
(DOCX)

S5 Fig Competing risk analysis between nSBP and w/o SBP patients considering death and liver transplantation during follow-up as competing risks. Death and liver transplantation were treated as competing risks. P-value (black colour): Comparison of the probability for death between nSBP and w/o SBP patients. P-value (red colour): Comparison of the probability for liver transplantation between nSBP and w/o SBP patients.
(DOCX)

S6 Fig Mortality comparison between never SBP and nSBP resolved patients (analysis 2). P-value was calculated using the log-rank test.
(DOCX)

S7 Fig Competing risk analysis between nSBP resolved and never SBP patients considering death and liver transplantation during follow-up as competing risks. Death and liver transplantation were treated as competing risks. P-value (black colour): Comparison of the probability for death between nSBP resolved and never SBP patients. P-value (red colour): Comparison of the probability for liver transplantation between nSBP resolved and never SBP patients.
(DOCX)

S8 Fig Mortality comparison within the nSBP resolved patient cohort between patients with and without SBP recurrence within 90 days from the diagnosis of resolved nSBP. P-value was calculated using the log-rank test.
(DOCX)

S9 Fig Comparison of SBP recurrence rates within the nSBP resolved patient cohort between patients with and without secondary antibiotic prophylaxis. Quinolones and rifaximin were considered as secondary antibiotic prophylaxis. P-value was calculated using the log-rank test.
(DOCX)

S10 Fig Mortality comparison within the nSBP resolved patient cohort between patients with and without secondary antibiotic prophylaxis. Quinolones and rifaximin were considered as secondary antibiotic prophylaxis. P-value was calculated using the log-rank test.
(DOCX)

S1 Table Reasons for hospitalization and Re-admission within the analysis 1 cohort. More than one reason for hospitalization per patient is possible.
(DOCX)

S2 Table Risk factors for death in ACLF patients. Uni- and multivariate Cox-regression analysis within nSBP and w/o SBP patients that had ACLF at baseline. n.s.: not significant; CI: confidence interval; HR: Hazard Ratio.
(DOCX)

S3 Table Risk factors for death in no-ACLF patients. Uni- and multivariate Cox-regression analysis within nSBP and w/o SBP patients that did not have ACLF at baseline. n.s.: not significant; CI: confidence interval; HR: Hazard Ratio.
(DOCX)

S4 Table Distribution of death and liver transplantation (LTx) during follow-up throughout the entire study cohort and the subgroups. (DOCX)

S5 Table Numbers of patients within the groups and subgroups. (DOCX)

S6 Table Antibiotic prophylaxis in nSBP resolved patients. (DOCX)

S7 Table Risk factors for death in nSBP resolved patients. Uni- and multivariate Cox-regression analysis (Model 3) within nSBP resolved patients only also considering secondary antibiotic prophylaxis (quinolone antibiotics and rifaximin were considered) as a parameter. n.s.: not significant; CI: confidence interval; HR: Hazard Ratio.
(DOCX)

S8 Table Positive ascites cultures of SBP patients during hospitalization indicating the distribution of Gram positive, Gram negative and MDR bacteria. (DOCX)

S9 Table Individual list of detected bacterial species in positive ascites cultures of SBP patients during hospitalization. Percentages based on number of caSBP (n = 14) and nSBP (n = 74) patients with positive ascites cultures, respectively. Number of patients with positive ascites cultures with more than one detected bacterial species (nSBP n = 23; caSBP n = 5).
(DOCX)

## Abbreviations

ACLF: acute-on-chronic liver failure
INR: International normalized ratio
ALT: alanine transaminase
caSBP: community acquired spontaneous bacterial peritonitis
CRP: c-reactive protein
GGT: gamma glutamyl transferase
HE: Hepatic encephalopathy
HIV: human immunodeficiency virus
HR: hazard ratio
MELD: Model of End Stage Liver Disease
nSBP: nosocomial acquired spontaneous bacterial peritonitis
SBP: spontaneous bacterial peritonitis
w/o SBP: without spontaneous bacterial peritonitis
x ULN: times upper limit of normal
