# Supplementary material for: Sustained impact of nosocomial-acquired spontaneous bacterial peritonitis in different stages of decompensated liver cirrhosis
Source: PLoS One. 2019 Aug 2;14(8):e0220666. doi: 10.1371/journal.pone.0220666 (PMC6677299; doi:10.1371/journal.pone.0220666)
Supplement: S1 Fig — P-value was calculated using the log-rank test. p1 compares w/o SBP and nSBP, p2 compares w/o SBP and caSBP. (DOCX) [file pone.0220666.s002.docx]

## S1 Fig: Mortality comparison within the overall cohort (analysis 1).

P-value was calculated using the log-rank test. p_1_ compares w/o SBP and nSBP, p_2_ compares w/o SBP and caSBP.

**

**
