# Supplementary material for: Sustained impact of nosocomial-acquired spontaneous bacterial peritonitis in different stages of decompensated liver cirrhosis
Source: PLoS One. 2019 Aug 2;14(8):e0220666. doi: 10.1371/journal.pone.0220666 (PMC6677299; doi:10.1371/journal.pone.0220666)
Supplement: S2 Fig — P-value was calculated using the log-rank test. (DOCX) [file pone.0220666.s003.docx]

## S2 Fig: Mortality comparison between patients with and without at least grade 1 ACLF at baseline within the overall cohort (analysis 1).

P-value was calculated using the log-rank test.
