# Supplementary material for: Sustained impact of nosocomial-acquired spontaneous bacterial peritonitis in different stages of decompensated liver cirrhosis
Source: PLoS One. 2019 Aug 2;14(8):e0220666. doi: 10.1371/journal.pone.0220666 (PMC6677299; doi:10.1371/journal.pone.0220666)
Supplement: S5 Fig — Death and liver transplantation were treated as competing risks. P-value (black colour): Comparison of the probability for death between nSBP and w/o SBP patients. P-value (red colour): Comparison of the probability for liver transplantation between nSBP and w/o SBP patients. (DOCX) [file pone.0220666.s006.docx]

## S5 Fig: Competing risk analysis between nSBP and w/o SBP patients considering death and liver transplantation during follow-up as competing risks.

Death and liver transplantation were treated as competing risks.
P-value (black colour): Comparison of the probability for death between nSBP and w/o SBP patients.
P-value (red colour): Comparison of the probability for liver transplantation between nSBP and w/o SBP patients.

**
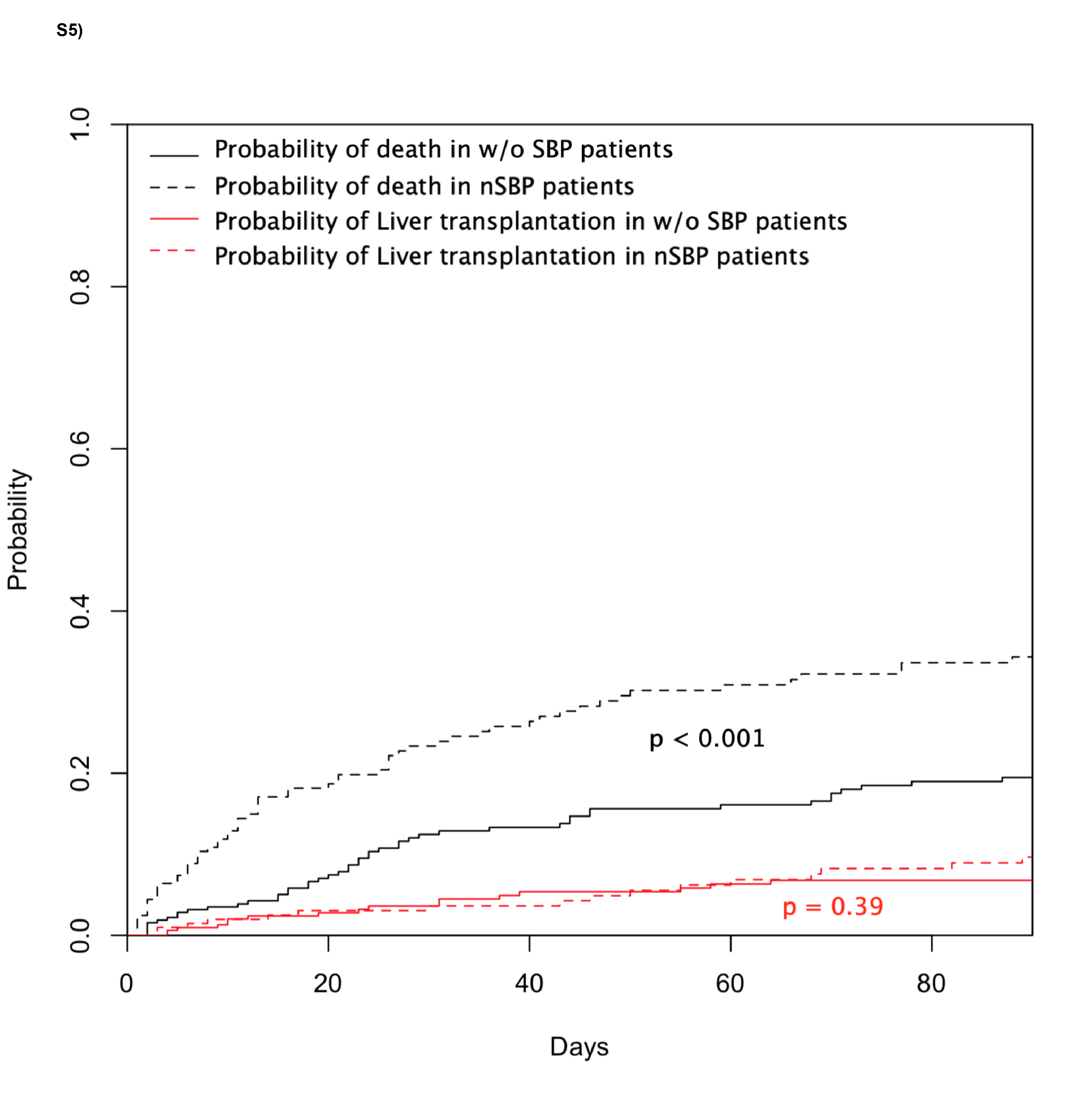
**
