# Supplementary material for: Sustained impact of nosocomial-acquired spontaneous bacterial peritonitis in different stages of decompensated liver cirrhosis
Source: PLoS One. 2019 Aug 2;14(8):e0220666. doi: 10.1371/journal.pone.0220666 (PMC6677299; doi:10.1371/journal.pone.0220666)
Supplement: S8 Fig — P-value was calculated using the log-rank test. (DOCX) [file pone.0220666.s009.docx]

## S8 Fig: Mortality comparison within the nSBP resolved patient cohort between patients with and without SBP recurrence within 90 days from the diagnosis of resolved nSBP.

P-value was calculated using the log-rank test.
