# Supplementary material for: Sustained impact of nosocomial-acquired spontaneous bacterial peritonitis in different stages of decompensated liver cirrhosis
Source: PLoS One. 2019 Aug 2;14(8):e0220666. doi: 10.1371/journal.pone.0220666 (PMC6677299; doi:10.1371/journal.pone.0220666)
Supplement: S10 Fig — Quinolones and rifaximin were considered as secondary antibiotic prophylaxis. P-value was calculated using the log-rank test. (DOCX) [file pone.0220666.s011.docx]

## S10 Fig: Mortality comparison within the nSBP resolved patient cohort between patients with and without secondary antibiotic prophylaxis.

Quinolones and rifaximin were considered as secondary antibiotic prophylaxis**.**P-value was calculated using the log-rank test.

**

**
