# Supplementary material for: Sustained impact of nosocomial-acquired spontaneous bacterial peritonitis in different stages of decompensated liver cirrhosis
Source: PLoS One. 2019 Aug 2;14(8):e0220666. doi: 10.1371/journal.pone.0220666 (PMC6677299; doi:10.1371/journal.pone.0220666)
Supplement: S1 Table — More than one reason for hospitalization per patient is possible. (DOCX) [file pone.0220666.s012.docx]

## S1 Table: Reasons for hospitalization and Re-admission within the analysis 1 cohort. More than one reason for hospitalization per patient is possible.

| **Reasons for hospitalization** | **Liver related** | **Ascites** | **GI-bleeding** | **TIPS/TIPS-Evaluation** | **LTx/LTx-Evaluation** | **Further diagnostics** | **Unplanned Readmission during follow up** |
| --- | --- | --- | --- | --- | --- | --- | --- |
| **Analysis 1 cohort, n** | 558 | 480 | 28 | 88 | 79 | 33 | 154 |
| **w/o SBP, n** | 308 | 254 | 17 | 54 | 40 | 20 | 57 |
| **caSBP, n** | 55 | 51 | 1 | 7 | 4 | 5 | 22 |
| **nSBP, n** | 195 | 175 | 10 | 27 | 35 | 8 | 75 |
