# Supplementary material for: Sustained impact of nosocomial-acquired spontaneous bacterial peritonitis in different stages of decompensated liver cirrhosis
Source: PLoS One. 2019 Aug 2;14(8):e0220666. doi: 10.1371/journal.pone.0220666 (PMC6677299; doi:10.1371/journal.pone.0220666)
Supplement: S2 Table — Uni- and multivariate Cox-regression analysis within nSBP and w/o SBP patients that had ACLF at baseline. n.s.: not significant; CI: confidence interval; HR: Hazard Ratio. (DOCX) [file pone.0220666.s013.docx]

## S2 Table: Risk factors for death in ACLF patients.

Uni- and multivariate Cox-regression analysis (model 1) within nSBP and w/o SBP patients that had ACLF at baseline. n.s.: not significant; CI: confidence interval; HR: Hazard Ratio.

| Risk factors for death | UnivariateHR | 95% CI | p-value | Multivariate Adjusted HR | 95% CI | p-value |
| --- | --- | --- | --- | --- | --- | --- |
| nSBP (yes) | 1.73 | 1.08-2.77 | 0.02 | 1.57 | 0.98-2.51 | **0.06** |
| MELD | 1.12 | 1.08-1.16 | <0.001 | 1.08 | 1.08-1.15 | **<0.001** |
| ALT (x ULN) | 1.01 | 0.98-1.04 | 0.57 |  |  |  |
| Gender (Male) | 1.03 | 0.64-1.65 | 0.92 |  |  |  |
| Age (years) | 0.99 | 0.97-1.01 | 0.52 |  |  |  |
| Platelets | 0.997 | 0.99-1.00 | 0.06 |  |  |  |
| Sodium | 0.98 | 0.95.1.01 | 0.26 |  |  |  |
| GGT (x ULN) | 0.96 | 0.89-1.03 | 0.21 |  |  |  |
