# Supplementary material for: Sustained impact of nosocomial-acquired spontaneous bacterial peritonitis in different stages of decompensated liver cirrhosis
Source: PLoS One. 2019 Aug 2;14(8):e0220666. doi: 10.1371/journal.pone.0220666 (PMC6677299; doi:10.1371/journal.pone.0220666)
Supplement: S3 Table — Uni- and multivariate Cox-regression analysis within nSBP and w/o SBP patients that did not have ACLF at baseline. n.s.: not significant; CI: confidence interval; HR: Hazard Ratio. (DOCX) [file pone.0220666.s014.docx]

## S3 Table: Risk factors for death in no-ACLF patients.

Uni- and multivariate Cox-regression analysis (model 1) within nSBP and w/o SBP patients that did not have ACLF at baseline. n.s.: not significant; CI: confidence interval; HR: Hazard Ratio.

| Risk factors for death | UnivariateHR | 95% CI | p-value | Multivariate Adjusted HR | 95% CI | p-value |
| --- | --- | --- | --- | --- | --- | --- |
| nSBP (yes) | 2.04 | 1.08-3.86 | 0.03 | 1.64 | 0.86-3.14 | 0.14 |
| MELD | 1.15 | 1.09-1.21 | <0.001 | 1.18 | 1.12-1.25 | **<0.001** |
| ALT (x ULN) | 1.03 | 0.88-1.20 | 0.73 |  |  |  |
| Gender (Male) | 1.36 | 0.70-2.63 | 0.36 |  |  |  |
| Age (years) | 1.03 | 0.998-1.05 | 0.07 | 1.05 | 1.02-1.08 | **<0.01** |
| Platelets | 1.00 | 0.998-1.004 | 0.61 |  |  |  |
| Sodium | 1.01 | 0.95-1.08 | 0.69 |  |  |  |
| GGT (x ULN) | 1.05 | 0.98-1.13 | 0.16 |  |  |  |
