# Supplementary material for: Sustained impact of nosocomial-acquired spontaneous bacterial peritonitis in different stages of decompensated liver cirrhosis
Source: PLoS One. 2019 Aug 2;14(8):e0220666. doi: 10.1371/journal.pone.0220666 (PMC6677299; doi:10.1371/journal.pone.0220666)
Supplement: S4 Table — (DOCX) [file pone.0220666.s015.docx]

## S4 Table: Distribution of death and liver transplantation (LTx) during follow-up throughout the entire study cohort and the subgroups.

| **Death or LTx during follow-up** | **MELD <15** | **MELD 15-25** | **MELD >25** | **All MELD-scores** |
| --- | --- | --- | --- | --- |
| Ana 1 cohort death, n (%) | 13 (6.53) | 42 (17.95) | 63 (43.15) | 118 (20.38) |
| Ana 1 cohort LTx, n (%) | 0 (0.00) | 8 (4.02) | 26 (17.81) | 34 (5.87) |
| w/o SBP death, n (%) | 7 (5.47) | 15 (12.10) | 26 (36.11) | 48 (15.00) |
| w/o SBP LTx, n (%) | 0 (0.00) | 3 (2.42) | 14 (19.44) | 17 (5.31) |
| caSBP death, n (%) | 1 (4.76) | 3 (13.04) | 3 (25.00) | 7 (12.50) |
| caSBP LTx, n (%) | 0 (0.00) | 1 (4.35) | 0 (0.00) | 1 (1.79) |
| nSBP death, n (%) | 5 (9.26) | 24 (27.59) | 34 (54.83) | 63 (31.03) |
| nSBP LTx, n (%) | 0 (0.00) | 4 (4.60) | 12 (19.35) | 16 (7.88) |
| Ana 2 cohort death, n (%) | 9 (6.34) | 28 (17.28) | 38 (42.70) | 75 (19.08) |
| Ana 2 cohort LTx, n (%) | 0 (0.00) | 6 (3.70) | 15 (16.85) | 21 (5.34) |
| Never SBP death, n (%) | 7 (6.25) | 13 (11.71) | 23 (37.70) | 43 (15.14) |
| Never SBP LTx, n(%) | 0 (0.00) | 2 (1.80) | 11 (18.03) | 13 (4.58) |
| nSBP resolved death, n (%) | 2 (6.66) | 15 (29.41) | 15 (53.57) | 32 (29.36) |
| nSBP resolved LTx, n (%) | 0 (0.00) | 4 (7.84) | 4 (14.29) | 8 (7.34) |
