# Supplementary material for: Sustained impact of nosocomial-acquired spontaneous bacterial peritonitis in different stages of decompensated liver cirrhosis
Source: PLoS One. 2019 Aug 2;14(8):e0220666. doi: 10.1371/journal.pone.0220666 (PMC6677299; doi:10.1371/journal.pone.0220666)
Supplement: S5 Table — (DOCX) [file pone.0220666.s016.docx]

## S5 Table: Numbers of patients within the groups and subgroups.

|  | **w/o SBP** | **caSBP** | **nSBP** | **never SBP** | **nSBP resolved** |
| --- | --- | --- | --- | --- | --- |
| MELD <15 | 124 | 21 | 54 | 112 | 30 |
| MELD 15-25 | 124 | 23 | 87 | 111 | 51 |
| MELD >25 | 72 | 12 | 62 | 61 | 28 |
| All MELD-scores | 320 | 56 | 203 | 284 | 109 |
