# Supplementary material for: Sustained impact of nosocomial-acquired spontaneous bacterial peritonitis in different stages of decompensated liver cirrhosis
Source: PLoS One. 2019 Aug 2;14(8):e0220666. doi: 10.1371/journal.pone.0220666 (PMC6677299; doi:10.1371/journal.pone.0220666)
Supplement: S6 Table — (DOCX) [file pone.0220666.s017.docx]

## S6 Table: Antibiotic prophylaxis in nSBP resolved patients.

| **Secondary antibiotic prophylaxis in nSBP resolved patients** | **nSBP resolved (n=109)** |
| --- | --- |
| Rifaximin, n (%) | 32 (29.36) |
| Norfloxacin, n (%) | 47 (43.12) |
| Fluoroquinolones, n (%) | 48 (44.04) |
| At least one secondary antibiotic prophylaxis, n (%) | 67 (61.47) |
