# Supplementary material for: Sustained impact of nosocomial-acquired spontaneous bacterial peritonitis in different stages of decompensated liver cirrhosis
Source: PLoS One. 2019 Aug 2;14(8):e0220666. doi: 10.1371/journal.pone.0220666 (PMC6677299; doi:10.1371/journal.pone.0220666)
Supplement: S7 Table — Uni- and multivariate Cox-regression analysis (Model 3) within nSBP resolved patients only also considering secondary antibiotic prophylaxis (quinolone antibiotics and rifaximin were considered) as a parameter. n.s.: not significant; CI: confidence interval; HR: Hazard Ratio. (DOCX) [file pone.0220666.s018.docx]

## S7 Table: Risk factors for death in nSBP resolved patients.

Uni- and multivariate Cox-regression analysis (Model 3) within nSBP resolved patients only also considering secondary antibiotic prophylaxis (quinolone antibiotics and rifaximin were considered) as a parameter. n.s.: not significant; CI: confidence interval; HR: Hazard Ratio.

| Risk factors for death | UnivariateHR | 95% CI | p-value | Multivariate Adjusted HR | 95% CI | p-value |
| --- | --- | --- | --- | --- | --- | --- |
| Antibiotic prophylaxis (yes) | 0.40 | 0.20-0.81 | 0.01 | 0.45 | 0.22-0.92 | **0.03** |
| MELD | 1.09 | 1.05-1.14 | <0.001 | 1.08 | 1.04-1.13 | **<0.001** |
| ALT (x ULN) | 1.19 | 0.97-1.46 | 0.09 | 1.03 | 0.77-1.38 | 0.83 |
| Gender (Male) | 1.33 | 0.63-2.81 | 0.46 |  |  |  |
| Age (years) | 1.02 | 0.99-1.05 | 0.18 |  |  |  |
| Platelets | 0.998 | 0.993-1.003 | 0.45 |  |  |  |
| Sodium | 1.01 | 0.95-1.07 | 0.69 |  |  |  |
| GGT (x ULN) | 1.02 | 0.98-1.07 | 0.34 |  |  |  |
