# Supplementary material for: Sustained impact of nosocomial-acquired spontaneous bacterial peritonitis in different stages of decompensated liver cirrhosis
Source: PLoS One. 2019 Aug 2;14(8):e0220666. doi: 10.1371/journal.pone.0220666 (PMC6677299; doi:10.1371/journal.pone.0220666)
Supplement: S8 Table — (DOCX) [file pone.0220666.s019.docx]

## S8 Table: Positive ascites cultures of SBP patients during hospitalization indicating the distribution of Gram positive, Gram negative and MDR bacteria.

| Bacteria positive ascites cultures of SBP patients during hospitalization | All SBP patients (n=259) | caSBP (n=56) | nSBP (n=203) |
| --- | --- | --- | --- |
| Gram positive, n (%) | 60 (23.17) | 11 (19.64) | 49 (24.14) |
| Gram negative, n (%) | 28 (10.81) | 3 (5.36) | 25 (12.32) |
| MDR, n (%) | 5 (1.93) | 0 (0.00) | 5 (2.46) |
