# Supplementary material for: Sustained impact of nosocomial-acquired spontaneous bacterial peritonitis in different stages of decompensated liver cirrhosis
Source: PLoS One. 2019 Aug 2;14(8):e0220666. doi: 10.1371/journal.pone.0220666 (PMC6677299; doi:10.1371/journal.pone.0220666)
Supplement: S9 Table — Percentages based on number of caSBP (n = 14) and nSBP (n = 74) patients with positive ascites cultures, respectively. Number of patients with positive ascites cultures with more than one detected bacterial species (nSBP n = 23; caSBP n = 5). (DOCX) [file pone.0220666.s020.docx]

## S9 Table: Individual list of detected bacterial species in positive ascites cultures of SBP patients during hospitalization.

Percentages based on number of caSBP (n=14) and nSBP (n=74) patients with positive ascites cultures, respectively. Number of patients with positive ascites cultures with more than one detected bacterial species (nSBP n=23; caSBP n=5).

| Detected bacterial species in positive ascites cultures of SBP patients during hospitalization | caSBP (n=56) | nSBP (n=203) |
| --- | --- | --- |
| Acinetobacter baumanii, n (%) | 0 (0.00) | 1 (1.35) |
| Acinetobacter sp., n (%) | 0 (0.00) | 1 (1.35) |
| Acinetobacter iwoffii, n (%) | 0 (0.00) | 1 (1.35) |
| Achromobacter xylosoxidans, n (%) | 0 (0.00) | 1 (1.35) |
| Bacillus sp., n (%) | 0 (0.00) | 2 (2.70) |
| Bacteriodes uniformis, n (%) | 0 (0.00) | 1 (1.35) |
| Corynebacterium amycolatum, n (%) | 0 (0.00) | 1 (1.35) |
| Corynebacterium jeikeium, n (%) | 0 (0.00) | 1 (1.35) |
| Corynebacterium minutissimum, n (%) | 1 (7.14) | 0 (0.00) |
| Enterobacter cloacae, n (%) | 0 (0.00) | 1 (1.35) |
| Enterococcus avium, n (%) | 1 (7.14) | 0 (0.00) |
| Enterococcus casseliflavus, n (%) | 0 (0.00) | 1 (1.35) |
| Enterococcus cecorum, n (%) | 0 (0.00) | 1 (1.35) |
| Enterococcus faecalis, n (%) | 1 (7.14) | 8 (10.81) |
| Enterococcus faecium, n (%) | 2 (14.29) | 10 (13.51) |
| Enterococcus faecium (VRE), n (%) | 0 (0.00) | 1 (1.35) |
| Escherichia coli, n (%) | 1 (7.14) | 9 (12.16) |
| Escherichia coli (3MRGN), n (%) | 0 (0.00) | 2 (2.70) |
| Klebsiella ornithiolytica, n (%) | 0 (0.00) | 1 (1.35) |
| Klebsiella oxytoca, n (%) | 1 (7.14) | 3 (4.05) |
| Klebsiella pneumoniae, n (%) | 0 (0.00) | 4 (5.41) |
| Pseudomonas aeruginosa, n (%) | 0 (0.00) | 1 (1.35) |
| Pseudomonas stutzeri, n (%) | 1 (7.14) | 0 (0.00) |
| Rothia dentocariosa, n (%) | 0 (0.00) | 1 (1.35) |
| Staphylococcus aureus, n (%) | 1 (7.14) | 13 (17.57) |
| Staphylococcus aureus (MRSA), n (%) | 0 (0.00) | 2 (2.70) |
| Staphylococcus capitis, n (%) | 0 (0.00) | 1 (1.35) |
| Staphylococcus epidermidis, n (%) | 4 (28.57) | 11 (14.86) |
| Staphylococcus haemolyticus, n (%) | 2 (14.29) | 7 (9.46) |
| Staphylococcus hominis, n (%) | 0 (0.00) | 2 (2.70) |
| Staphylococcus xylosus, n (%) | 1 (7.14) | 0 (0.00) |
| Stenotrophomonas maltophilia, n (%) | 0 (0.00) | 3 (4.05) |
| Streptococcus agalactiae, n (%) | 0 (0.00) | 1 (1.35) |
| Streptococcus mitis, n (%) | 0 (0.00) | 3 (4.05) |
| Streptococcus parasanguinis, n (%) | 3 (21.43) | 0 (0.00) |
| Streptococcus sanguinis, n (%) | 1 (7.14) | 0 (0.00) |
| Streptococcus vestibularis, n (%) | 0 (0.00) | 1 (1.35) |
